# Bridging inflammation and venous thrombosis: the NLRP3 inflammasome connection

**DOI:** 10.3389/fcvm.2025.1584745

**Published:** 2025-05-30

**Authors:** Jian Wu, Zilong Wang, Wei Shao, Jianwen Mo

**Affiliations:** ^1^ The First Clinical College, Gannan Medical University, Ganzhou, Jiangxi, China; ^2^ Ganzhou Key Laboratory of Osteoporosis Research, The First Affiliated Hospital of Gannan Medical University, Ganzhou, Jiangxi, China; ^3^ Department of Orthopedics, The First Affiliated Hospital of Gannan Medical University, Ganzhou, Jiangxi, China

**Keywords:** NLRP3 inflammasome, thrombosis, inflammation, IL-1β, NETs

## Abstract

NLRP3 (NACHT, LRR, and PYD domains-containing protein 3) inflammasome is a critical regulator of inflammatory responses in the body and is closely associated with the inflammatory processes of various diseases. In recent years, research has increasingly focused on the role of the NLRP3 inflammasome in venous thromboembolism (VTE). Venous thromboembolism is a common and potentially fatal vascular disease with a complex pathophysiology involving multiple cellular and molecular pathways. The NLRP3 inflammasome activates caspase-1 downstream, facilitating the maturation and secretion of pro-inflammatory cytokines such as IL-1β and IL-18, triggering local and systemic inflammatory responses. These inflammatory reactions can promote the recruitment and activation of immune cells (such as monocytes and neutrophils), platelet activation, endothelial cell damage, and aggregation, ultimately leading to thrombus formation. Additionally, the interaction of the NLRP3 inflammasome with the coagulation system further exacerbates the risk of thrombosis. In summary, the NLRP3 inflammasome plays a critical role in the development of venous thrombosis, and interventions targeting it may offer new insights and strategies for the prevention and treatment of venous thrombosis. This review provides an overview of the current understanding of how the NLRP3 inflammasome promotes venous thrombosis, highlighting recent preclinical research advancements and potential therapeutic agents in this field.

## 1 Introduction

Venous thromboembolism typically originates from the formation of deep vein thrombosis (DVT), with pulmonary embolism (PE) potentially progressing as a complication when emboli detach (1). The risk factors for DVT include major surgery, trauma, fractures, and prolonged immobility (2). Inflammation plays a crucial role in the formation of venous thrombosis (3). It is the body's protective response to infection or injury, involving processes such as vasodilation, immune cell recruitment, and activation of immune cells. This inflammatory response aids in the clearance of harmful stimuli and promotes tissue repair, highlighting the significance of inflammatory regulation in preventing infection and preserving tissue (4, 5). Inflammation acts as a defense mechanism against external stimuli, regulating immune responses by producing cytokines to facilitate pathogen clearance and tissue healing (6). However, an excessive immune response can lead to hypercoagulability (7, 8). The terms immunothrombosis (a physiological host defense mechanism) and thromboinflammation (a broader pathological interplay) are frequently used in discussing the link between inflammation and thrombosis, but they refer to distinct processes. Immunothrombosis describes the physiological process where the innate immune system interacts with the coagulation system to form a thrombus as a host defense mechanism, particularly at sites of infection or tissue damage (8). This process is generally protective but can become pathological if dysregulated. Thromboinflammation, on the other hand, refers to the broader interplay between inflammatory and thrombotic pathways, including both immunothrombosis and the involvement of inflammatory cells and cytokines in promoting a prothrombotic state (9). NLRP3 (NACHT-, LRR-, and pyrin domain-containing protein 3) is an innate immune receptor that senses exogenous or endogenous stimuli. When cells respond to stress signals, NLRP3 can assemble with other proteins to form the NLRP3 inflammasome, producing and releasing inflammatory factors such as IL-1β, IL-18, and inducing pyroptosis, a lytic cell death process that releases pro-inflammatory mediators (10–12). Activation of the NLRP3 inflammasome and its downstream signaling molecules can recruit and activate leukocytes and platelets while inducing endothelial dysfunction, leading to the release of various pro-inflammatory and pro-coagulant factors. This accelerates thromboinflammation and establishes a prothrombotic endothelial phenotype. Additionally, immune cells can release tissue factor (TF), and neutrophils can transform into neutrophil extracellular traps (NETs), which together promote thrombosis (11–13). Numerous preclinical studies have shown that inhibition of the NLRP3 inflammasome and IL-1 signaling pathway can effectively prevent venous thrombosis (14). These findings offer promise for the development of additional therapeutic strategies aimed at inhibiting the onset of DVT, reducing the risk of recurrence, and mitigating thrombotic complications (14).

## 2 Overview of the NLRP3 inflammasome pathway

The innate immune system serves as the body's first line of defense in detecting signals from external stimuli, thereby triggering an adaptive immune response. It adeptly identifies environmental irritants, infectious pathogens, and deceased cells, subsequently activating downstream signaling pathways that induce an inflammatory response, essential for eradicating infections and facilitating tissue repair. This system functions through germline-encoded pattern recognition receptors (PRRs), which enable it to swiftly discern and react to potential threats, ensuring the body's resilience against external challenges (15). PRRs have the ability to recognize both pathogen-associated molecular patterns (PAMPs) and damage-associated molecular patterns (DAMPs). These include a diverse array of molecules such as lipopolysaccharides, microbial nucleic acids, ATP, reactive oxygen species (ROS), pore-forming toxins, and various particles. Upon detection of these molecular patterns, PRRs are activated, initiating downstream signaling cascades that propagate signals and stimulate both innate and adaptive immune responses (16). PRRs are categorized based on their localization, with receptors binding either to the cell membrane or the cytoplasm. Representative examples include Toll-like receptors (TLRs), predominantly situated on the cell membrane, and NOD-like receptors (NLRs), primarily found in the cytoplasm (17). The NLR family members serve as crucial inflammasome sensors in the innate immune system, playing a pivotal role in pathogen recognition and inflammatory response regulation. Among them, NLRP3, NLRC4, and AIM2 all possess the ability to assemble inflammasomes but exhibit distinct recognition mechanisms: AIM2 initiates inflammatory responses by detecting cytosolic DNA (such as viral/bacterial DNA or aberrant host DNA) (18); NLRC4 specifically recognizes bacterial flagellin and type III secretion system proteins, playing a central defensive role in infections like Salmonella (19), and can cooperate with AIM2 to modulate inflammation (20); whereas NLRP3, as the most versatile environmental sensor, stands out due to its unique capacity to integrate multiple cellular stress signals.The production of reactive oxygen species (ROS) is a key signal for NLRP3 inflammasome activation. ROS promotes NLRP3 activation through various pathways, such as inducing lysosomal membrane permeabilization via oxidative stress, leading to the release of lysosomal enzymes like cathepsin B, which further enhances NLRP3 activation (21, 22). Additionally, mitochondrial dysfunction is closely linked to NLRP3 activation. Mitochondrial damage triggers excessive ROS production and increases mitochondrial membrane permeability, releasing pro-inflammatory factors that activate NLRP3 (23). Potassium (K^+^) efflux is another critical signal for NLRP3 inflammasome activation. A decrease in intracellular K^+^ concentration is considered a prerequisite for NLRP3 activation. Studies show that K^+^ efflux during NLRP3 activation can occur through multiple mechanisms, including ATP-activated P2X7 receptor channels and bacterial ionophores like nigericin (24). Lysosomal rupture is also a key step in NLRP3 inflammasome activation, as it releases lysosomal contents—including various enzymes and pro-inflammatory factors—into the cytoplasm, directly or indirectly promoting NLRP3 activation (25, 26). This multifaceted signal integration capability makes NLRP3 a central hub linking pathological stimuli to inflammatory responses.Moreover, the NLRP3 inflammasome exhibits highly conserved structure and function across species, indicating strong evolutionary preservation (27). This conservation extends not only to its structure but also to its responsiveness to diverse stimuli, establishing NLRP3 as a core sensor in sterile inflammation. The NLRP3 inflammasome plays a critical role in numerous sterile inflammatory diseases. For instance, in atherosclerosis, NLRP3 accelerates disease progression by promoting inflammation (28). Similarly, in acute kidney injury and chronic kidney disease, NLRP3 activation is closely associated with renal inflammation and fibrosis (29). In these diseases, aberrant NLRP3 activation can lead to tissue damage and functional impairment.In summary, stress signals such as ROS, mitochondrial dysfunction, K^+^ efflux, and lysosomal rupture interact through complex mechanisms to collectively regulate NLRP3 inflammasome activation. These integrated signals provide protective effects under physiological conditions while driving inflammatory responses in pathological states, highlighting their potential as therapeutic targets in disease treatment (23, 25, 30).

The assembly of the NLRP3 inflammasome involves three key components: NLRP3, ASC, and caspase-1. NLRP3 comprises a C-terminal leucine-rich repeat (LRR) domain, a central nucleotide-binding NACHT domain with ATPase activity, and an N-terminal pyrin domain (PYD). ASC contains a PYD and a C-terminal caspase activation and recruitment domain (CARD). Caspase-1 is composed of several structural elements, including a CARD and catalytic subunits referred to as p20 and p10. These catalytic subunits are crucial for the enzymatic activity of caspase-1, enabling it to cleave its substrate proteins and execute its cellular functions (10, 31–33). The activation of the NLRP3 inflammasome involves two distinct steps: priming and activation. During the initiation step, pattern recognition receptors detect extracellular or intracellular signals, leading to downstream signaling events that culminate in the activation of NF-κB. Activated NF-κB translocates to the nucleus, where it orchestrates the expression of various genes, including those encoding NLRP3, pro-IL-1β, and pro-IL-18. This transcriptional upregulation provides primes the cell by increasing the abundance of inflammasome pathway components in the cytoplasm (34–36). Additionally, priming signals induce post-translational modifications (PTMs) of NLRP3, such as ubiquitination mediated by the Zn2+-containing JAMM domain metalloproteinase BRCC3 and phosphorylation mediated by c-Jun N-terminal kinase (JNK1).These PTMs play crucial roles in modulating the activity and responsiveness of NLRP3 to activation signals (37–39).

NEK7 acts as a licensing factor for NLRP3 inflammasome activation by binding NLRP3 after K^+^ efflux and stabilizing its active conformation, thereby enabling oligomerization and ASC recruitment (32, 40). Mechanistically, NEK7 does not scaffold inflammasome components but instead couples K^+^ efflux to NLRP3 activation by maintaining its oligomerization-competent state (40). Studies show that NEK7-NLRP3 interaction specifically occurs after K^+^ efflux and is essential for ASC speck formation and caspase-1 activation (40, 41). Overall, the initiation step sets the stage for NLRP3 inflammasome activation by preparing the cell and modifying NLRP3, making it poised for activation in response to specific stimuli.

**Figure 1:**
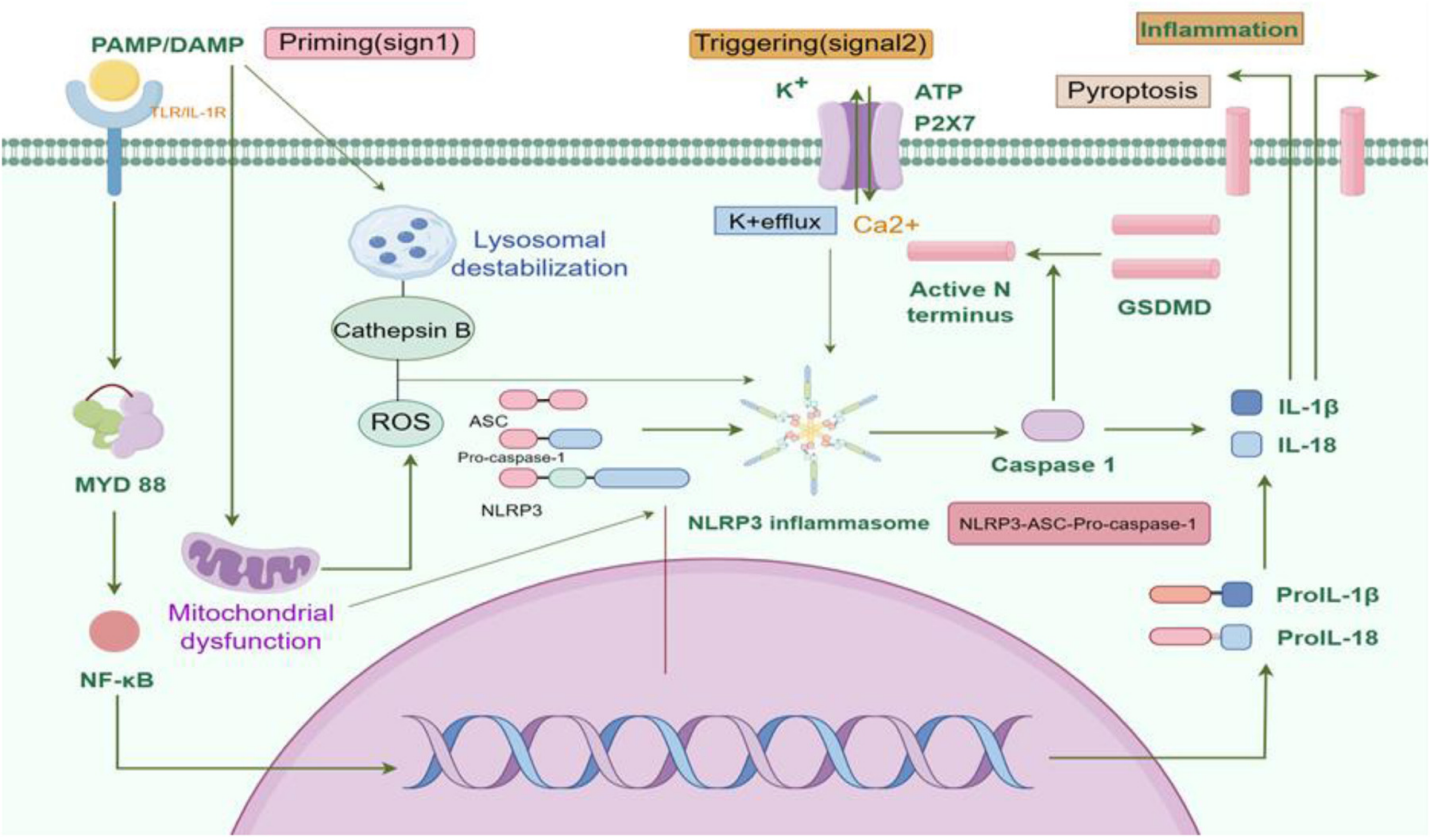
NLRP3 inflammasome signaling pathway. The activation of the NLRP3 inflammasome involves two signaling steps, namely the priming step and the activation step. In response to the stimulation of PAMPs and DAMPs, pattern recognition receptors receive stress signals and initiate the activation of NF-κB. This leads to the transportation of NF-κB into the nucleus, promoting the transcriptional expression of NLRP3, pro-IL-1β, pro-IL-18, and other inflammasome components. Accumulation of cytoplasmic substances prepares the basis for subsequent activation steps. Subsequently, under the stimulation of extracellular ATP, ROS, cathepsin B, calcium ions, lysosomal proteins, and mitochondrial proteins, the NLRP3 inflammasome assembles into the NLRP3-ASC-pro-caspase-1 complex. Pro-caspase-1 is then self-cleaved into active caspase-1, which cleaves pro-IL-1β, pro-IL-18, and GSDMD, leading to the activation of IL-1β, IL-18, and GSDMD. The N-terminal fragment of GSDMD translocates to the cell membrane, promoting the formation of membrane pores and cell pyroptosis. With the release of inflammatory factors such as IL-1β and IL-18, the occurrence and development of the inflammatory response are facilitated. AMPs, pathogen-associated molecular patterns; DAMPs, damage-associated molecular-patterns; NLRP3, NACHT-, LRR-, and pyrin domain-containing protein 3; IL-1β, Interleukin-1β; IL-18, Interleukin-18; ATP, Adenosine Triphosphate; ROS, reactive oxygen species; GSDMD, Gasdermin D. Created using Figdraw.

Activation signals for the NLRP3 inflammasome are triggered by PAMPs and DAMPs. These signals initiate a cascade of molecular and cellular events such as ion flux, lysosomal damage, reactive oxygen species, and mitochondrial dysfunction that ultimately lead to NLRP3 activation (Figure 1). These events are interconnected and can amplify each other, leading to robust NLRP3 activation (42). Initially, NLRP3 acts as a signaling hub, self-oligomerizing via NACHT domain interactions. Oligomerized NLRP3 then recruits ASC through matching PYD-PYD interactions. ASC, once recruited, polymerizes into filamentous structures, facilitating the recruitment and activation of caspase-1 via CARD-CARD domain interactions (43–45). The resulting assembly of NLRP3, ASC, and caspase-1 forms the NLRP3 inflammasome. Upon activation, pro-caspase-1 undergoes autocatalysis, producing active caspase-1. Active caspase-1 then cleaves pro-inflammatory cytokines such as IL-1β and IL-18 into their active forms, initiating an inflammatory response (35, 46, 47). Additionally, caspase-1 cleaves Gasdermin D (GSDMD), releasing its N-terminal domain. This domain translocates to the cell membrane, forming membrane pores that allow the release of intracellular contents, including IL-1β and IL-18. This process ultimately leads to pyroptosis (37, 48, 49). It is noteworthy that the secretion mechanisms of IL-1β and IL-18 are distinctly different from those of classical cytokines (e.g., IL-6, TNF-α). During pyroptosis, inflammasome activation induces Gasdermin D protein oligomerization to form plasma membrane pores, which directly mediate the release of these cytokines. This process does not rely on the conventional endoplasmic reticulum-Golgi secretory pathway, but rather achieves rapid, localized inflammatory signal propagation through plasma membrane rupture (50, 51).

## 3 Noncanonical and alternative inflammasome pathways

Noncanonical inflammasomes, also referred to as caspase-11-dependent inflammasomes in murine models, play a critical role in host defense against bacterial pathogens, particularly those containing lipopolysaccharide (LPS) on their outer membranes (52). In humans, caspases-4 and -5 serve analogous roles to caspase-11 in mice. When cells encounter LPS from Gram-negative bacteria, a series of events is triggered, primarily mediated by Toll-like receptor 4 (TLR4). Through the myeloid differentiation primary response 88 (MyD88)-dependent pathway, TLR4 activation induces the expression of pro-IL-1β precursor and NLRP3, contributing to priming of the cell for inflammasome activation (53). Simultaneously, LPS binding to TLR4 induces the expression of type I interferons, such as IFN-β, which further activate the TLR4 signaling pathway. This positive feedback loop leads to the upregulation of additional IFN-inducible genes, including pro-caspase-11 (54). Pro-caspase-11 is then cleaved and activated upon binding of intracellular LPS to its CARD, triggering its oligomerization and activation.Notably, caspases-4 and -5 in humans can similarly be activated by intracellular LPS binding to their CARD domains (55). Once activated, these caspases cleave GSDMD, leading to the formation of membrane pores, which cause potassium efflux, cellular swelling, and ultimately pyroptosis (56, 57). Importantly, pyroptosis induced by caspase-4/5/11 activation can also indirectly activate the canonical NLRP3 inflammasome by promoting potassium efflux, a known trigger for NLRP3 activation. This cascade culminates in the release of IL-1β and other pro-inflammatory cytokines, contributing to the host immune response against bacterial infection (58, 59).

## 4 Venous thrombosis formation: a multifactorial pathological process

Venous thrombosis formation is a complex pathological process driven by multiple factors, with its core mechanisms involving the interplay of Virchow's triad—venous stasis, hypercoagulability, and endothelial dysfunction (60). In the early stages, abnormal blood flow (e.g., hypoxic environments in venous valve sinuses) activates endothelial cells, leading to increased expression of adhesion receptors that promote the binding of circulating leukocytes and microvesicles (61). This activation further induces the release of TF, initiating the coagulation cascade (61). Concurrently, altered shear stress causes endothelial damage, triggering the formation of von Willebrand factor (VWF)-platelet aggregates resistant to ADAMTS13 cleavage, which serve as anchoring points for thrombus formation (62). Although venous thrombosis has traditionally been viewed as coagulation-dominated, studies reveal that platelets play a critical role through procoagulant activation: platelet factor 4 (PF4) promotes deep vein thrombosis by modulating NET formation (63), while cyclophilin D and transmembrane protein 16F deficiencies in murine models demonstrate the regulatory role of platelet activation pathways in thrombosis susceptibility (64). Platelet-derived extracellular vesicles, abundantly released post-trauma, not only contribute to hemostasis but also enhance thrombus formation (65).

At the immunoregulatory level, natural killer (NK) cells drive thrombus development by producing interferon-gamma (IFN-γ) to induce NET formation; NK cell depletion reduces NETs and attenuates thrombosis, highlighting their necessity (66). Neutrophils and erythrocytes exhibit heightened procoagulant activity under hypoxic conditions (e.g., high altitude or air travel), amplifying thrombotic risk (67). Molecularly, fibrin network architecture and factor XIII-mediated crosslinking determine thrombus mechanical stability (68), while elevated coagulation factor VIII and VWF levels are strongly associated with venous thrombosis risk (69), with VWF-mediated platelet adhesion shown to be essential in murine deep vein thrombosis models (70).

Notably, inflammatory responses permeate the entire thrombotic process: they promote thrombosis through cytokine networks while influencing thrombus resolution via fibrinolytic regulation and venous remodeling (71). These multilayered interactions provide a theoretical foundation for developing comprehensive therapeutic strategies targeting hemodynamics, cellular crosstalk, and molecular pathways.

## 5 Innate immune cells and venous thrombosis

**Figure 2:**
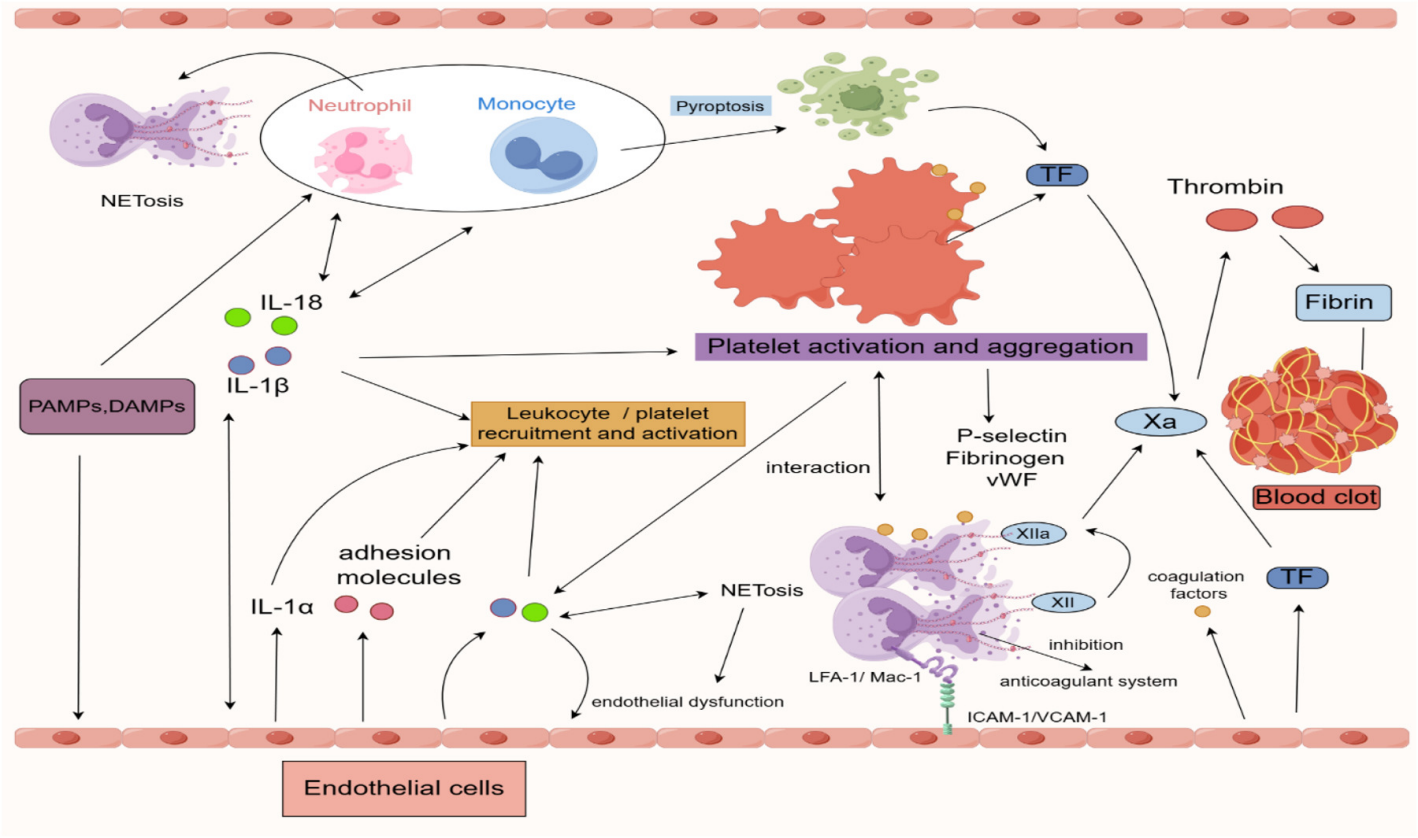
Inflammatory regulation in the process of venous thrombosis formation. IL-1α is released at sites of vascular injury, acting as a signaling molecule to recruit neutrophils, macrophages, and platelets. Macrophages are among the primary cells that activate the NLRP3 inflammasome, which leads to the maturation and secretion of pro-IL-1β. IL-1β, as a key pro-inflammatory cytokine, further activates other immune cells, including neutrophils and endothelial cells. Macrophages can express TF, a critical initiator of the extrinsic coagulation pathway, which activates the downstream coagulation cascade, thus promoting clot formation. Once activated, neutrophils release NETs, which can trap pathogens and aggregate clotting factors, enhancing thrombus formation. Neutrophils are also capable of expressing the NLRP3 inflammasome and can release IL-1 via autocrine and paracrine mechanisms, thereby further amplifying the local inflammatory response. The adhesion molecules expressed by neutrophils, such as LFA-1 and Mac-1, bind to ICAM-1 and VCAM-1 on endothelial cells, facilitating neutrophil adhesion and migration to the site of inflammation. Activated platelets release a variety of granules containing pro-inflammatory and pro-coagulant factors, such as P-selectin, fibrinogen, and vWF. Platelets can also expose TF on their surface, further promoting the coagulation cascade. Additionally, platelets provide a phospholipid surface that enhances the activity of clotting factors. Endothelial cells, under inflammatory stimuli, can express the NLRP3 inflammasome and secrete IL-1β, thereby contributing to the local inflammatory response. This process induces endothelial dysfunction and increases endothelial permeability. During inflammation, endothelial cells upregulate various adhesion molecules to promote leukocyte and platelet adhesion and migration. Moreover, endothelial cells can enhance the expression of pro-coagulant factors such as TF and vWF during the inflammatory process. PAMPs, pathogen-associated molecular-patterns; DAMPs, damage-associated molecular-patterns; IL, interleukin; NETs, neutrophil extracellular traps; NLRP3, NACHT-, LRR- and pyrin domain-containing protein 3; TF, tissue factor; VWF, von Willebrand factor, LFA-1/Mac-1, Lymphocyte Function-associated Antigen 1/Macrophage-1 Antigen; ICAM-1/VCAM-1, Intercellular Adhesion Molecule 1/Vascular Cell Adhesion Molecule 1. Created using Figdraw.

In the early stages of thrombus formation, the vascular injury site releases IL-1*α* as a signaling molecule to recruit neutrophils, macrophages, and platelets. These cells subsequently release mature inflammatory cytokines, IL-1β and IL-18 (14). IL-1β binds to IL-1R1 and recruits IL-1RAcP to form a complex, initiating the MyD88-dependent signaling pathway, activating IRAK/TRAF6, leading to IκB degradation and NF-κB nuclear translocation, thereby driving pro-inflammatory gene expression (20–22). IL-18 triggers a similar pathway via IL-18R (72). Together, they synergistically amplify the inflammatory response and promote thromboinflammation.The action of IL-1β is highly localized, primarily acting on neighboring cells in a paracrine manner to drive local inflammation amplification and tissue repair (73). IL-1β promotes endothelial cell expression of adhesion molecules (e.g., VCAM-1 and ICAM-1) and chemokines (e.g., MCP-1), enhancing leukocyte adhesion and migration, thereby exacerbating inflammation (74). Additionally, IL-1β promote the formation of NETs, aggravating inflammatory responses and endothelial dysfunction (75). The secretion of IL-1β and IL-18 ultimately facilitates leukocyte and platelet recruitment, increases vascular permeability, exacerbates inflammation, and promotes venous thrombosis (Figure 2) (14, 76, 77).

Monocytes and macrophages play a crucial role in inflammatory responses. Their activation leads to the release of various inflammatory factors, exacerbating inflammation and promoting venous thrombosis. Studies show that nuclear factor kappa B (NF-κB) acts as a master regulator of inflammation in monocytes and macrophages, modulating the expression of multiple chemokines, cytokines, and transcription factors, thereby playing a key role in inflammatory and immune responses (78). Inflammasome activation is also recognized as a significant contributor to venous thrombosis, particularly through pyroptosis (79). Activation of the NLRP3 inflammasome in monocytes and macrophages triggers pyroptosis, releasing pro-inflammatory cytokines such as IL-1β and IL-18, while also upregulating and secreting TF. TF is a key component of the extrinsic coagulation pathway, and its increased expression directly promotes venous thrombosis formation (80, 81). In infectious inflammation, LPS in the macrophage cytoplasm can activate caspase-11-dependent pyroptosis, ultimately triggering a lethal inflammatory response, releasing TF, IL-1β, and IL-18, thereby promoting coagulation (82). The role of monocytes and macrophages in thrombus formation extends beyond inflammatory responses, as they also regulate coagulation processes through interactions with platelets. Studies have revealed that the formation of monocyte-platelet aggregates can induce a pro-inflammatory phenotype in monocytes, thereby exacerbating thrombus formation (83).

Neutrophils can also form NETs, which play a critical role in thrombus development (76). NET formation begins with Peptidyl arginine deiminase 4(PAD4) activation, followed by chromatin decondensation, nuclear membrane rupture, and eventual plasma membrane disintegration (84). PAD4 is essential for NET formation and thrombosis induction (85). NETs can capture TF, promoting local thrombin generation and platelet activation, thereby facilitating thrombus formation (76, 86). Components of NETs, such as extracellular DNA, can bind to vWF and fibrin, providing a substrate for platelet adhesion and potentially activating FXII to drive coagulation (81, 87, 88). Moreover, fibrin coagulation in the presence of histone-DNA complexes enhances fibrin thickness, stiffness, and clot stability while prolonging clot lysis time (89). Beyond DNA, histones in NETs also contribute to coagulation. When NET-associated histones are released into the extracellular space, they bind to TLR2 or TLR4 on various cell surfaces, inducing cytotoxicity, pro-inflammatory cytokine production, and thrombosis (90). Extracellular histones, as endogenous molecules released by inflammatory or necrotic cells, exert potent procoagulant effects by activating platelets through multiple pathways. Studies indicate that histones primarily induce a procoagulant phenotype and promote thrombin generation by binding to platelet surface TLR2/TLR4 receptors, a process that can be inhibited by specific monoclonal antibodies (91). Furthermore, individual recombinant human histones (H1, H2A, H2B, H3, and H4) have been shown to serve as adhesive substrates, promoting platelet activation and aggregation, triggering fibrinogen binding and vWF release, with histone H4 exhibiting the most potent procoagulant activity (92). Further research reveals that signaling pathways such as ERK, Akt, p38, and NF-κB are involved in histone-mediated platelet activation, and inhibitors targeting these pathways significantly suppress platelet activation (92). Histones are highly cytotoxic and can directly damage endothelial cells by increasing membrane permeability, leading to membrane rupture and cell death, thereby compromising vascular integrity, inducing endothelial dysfunction, increasing vascular permeability, and promoting inflammation (88, 93). Additionally, histones and other molecules on NETs can activate endothelial cells, stimulating the secretion of pro-inflammatory cytokines such as IL-6, IL-8, and TNF-α, exacerbating local inflammation. By binding to endothelial cell surface receptors (e.g., TLR4), NETs can also activate inflammatory signaling pathways like NF-κB, promoting leukocyte recruitment and amplifying the inflammatory cascade (94, 95). NETs further enhance thrombin generation in plasma by activating the intrinsic coagulation pathway (91). Enzymes such as elastase and myeloperoxidase (MPO) in NETs degrade anticoagulants and tissue factor inhibitors, neutralizing endogenous anticoagulants and promoting thrombin formation (8, 14, 96).

NET formation may be linked to NLRP3 inflammasome activation in neutrophils. Studies show that PAD4 supports NLRP3 inflammasome assembly, activating the canonical NLRP3 inflammasome pathway, leading to caspase-1 activation, inducing neutrophil NETosis, a regulated form of neutrophil cell death characterized by the release of NETs, and promoting venous thrombosis in mice. This research particularly highlights NET formation under sterile conditions—where the NLRP3 inflammasome is activated by endogenous stimuli such as urate crystals or ATP, subsequently cooperating with PAD4 to drive NET formation (97). In another study, Chen et al. proposed the role of non-canonical inflammasome signaling in NET formation, emphasizing the critical function of GSDMD in this process. GSDMD forms membrane pores upon cleavage, triggering pyroptosis and promoting NET release (98). Unlike the previous study, this occurs under both infectious and sterile inflammatory conditions, where the non-canonical inflammasome (primarily caspase-11) is activated. Caspase-11 directly recognizes intracellular LPS, triggering GSDMD cleavage. Activated GSDMD forms pores in the cell membrane, leading to the release of nucleic acids and enzymes, ultimately resulting in NET formation (98). Future studies could compare infectious and sterile inflammation models to explore the dominant roles of these two pathways in different immune responses. For example, animal models could be used to selectively inhibit PAD4 and caspase-11, observing NET formation and analyzing the relative contributions of these pathways in different inflammatory contexts. New clinical trials could evaluate the effects of PAD4 inhibitors, caspase-11 inhibitors, or gasdermin D inhibitors in treating sterile and infectious inflammatory diseases, further investigating their impact on NET formation, inflammatory responses, and tissue damage to provide novel therapeutic insights for immune-mediated diseases. Thus, further research is needed to elucidate the underlying mechanisms of NLRP3 inflammasome-induced NETosis in neutrophils.

The interplay between NETosis and the NLRP3 inflammasome has also been documented. NETs can activate the canonical NLRP3 inflammasome pathway, enhancing caspase-1 expression and the release of IL-1β and IL-18 in macrophages, while IL-18 can, in turn, stimulate NETosis (99). Moreover, NLRP3-deficient mice exhibit reduced NETosis and attenuated venous thrombosis (97).

## 6 Platelets and venous thrombosis

Activation of the NLRP3 inflammasome in platelets drives the release of pro-inflammatory cytokines such as IL-1β, IL-18 and IL-1α (14), amplifying vascular inflammation, endothelial activation, and platelet aggregation—key processes in thrombosis (100–102). NLRP3-mediated IL-1β secretion further enhances platelet activation via autocrine signaling, though its role in sterile inflammation requires further investigation (103). Studies in NLRP3-deficient mice demonstrate reduced platelet aggregation, diminished integrin αIIbβ3 activation, and attenuated thrombus burden, highlighting NLRP3's regulatory role in platelet activation and thrombogenesis (104). NLRP3 inhibition (e.g., via MCC950) or genetic deletion reduces IL-1β-driven platelet hyperactivation in sepsis and LPS-induced models, emphasizing its therapeutic potential (105). Platelets and NETs synergistically promote intravascular coagulation. NET removal decreases thrombin activity and platelet aggregation probability (106). Notably, NLRP3 cooperates with Bruton's tyrosine kinase (BTK) to amplify platelet aggregation and thrombosis, particularly in diseases like sickle cell anemia (SCD), where HMGB1/TLR4-NLRP3/BTK crosstalk exacerbates thrombosis (107–109). Intriguingly, NLRP3 also influences thrombosis through inflammasome-independent mechanisms. Chen et al. revealed that NLRP3 deficiency impairs platelet aggregation and thrombus formation via GPIb-IX complex modulation, independent of IL-1 signaling (110). NLRP3's dual roles—both canonical and non-canonical—position it as a multifaceted therapeutic target.

**Figure 3:**
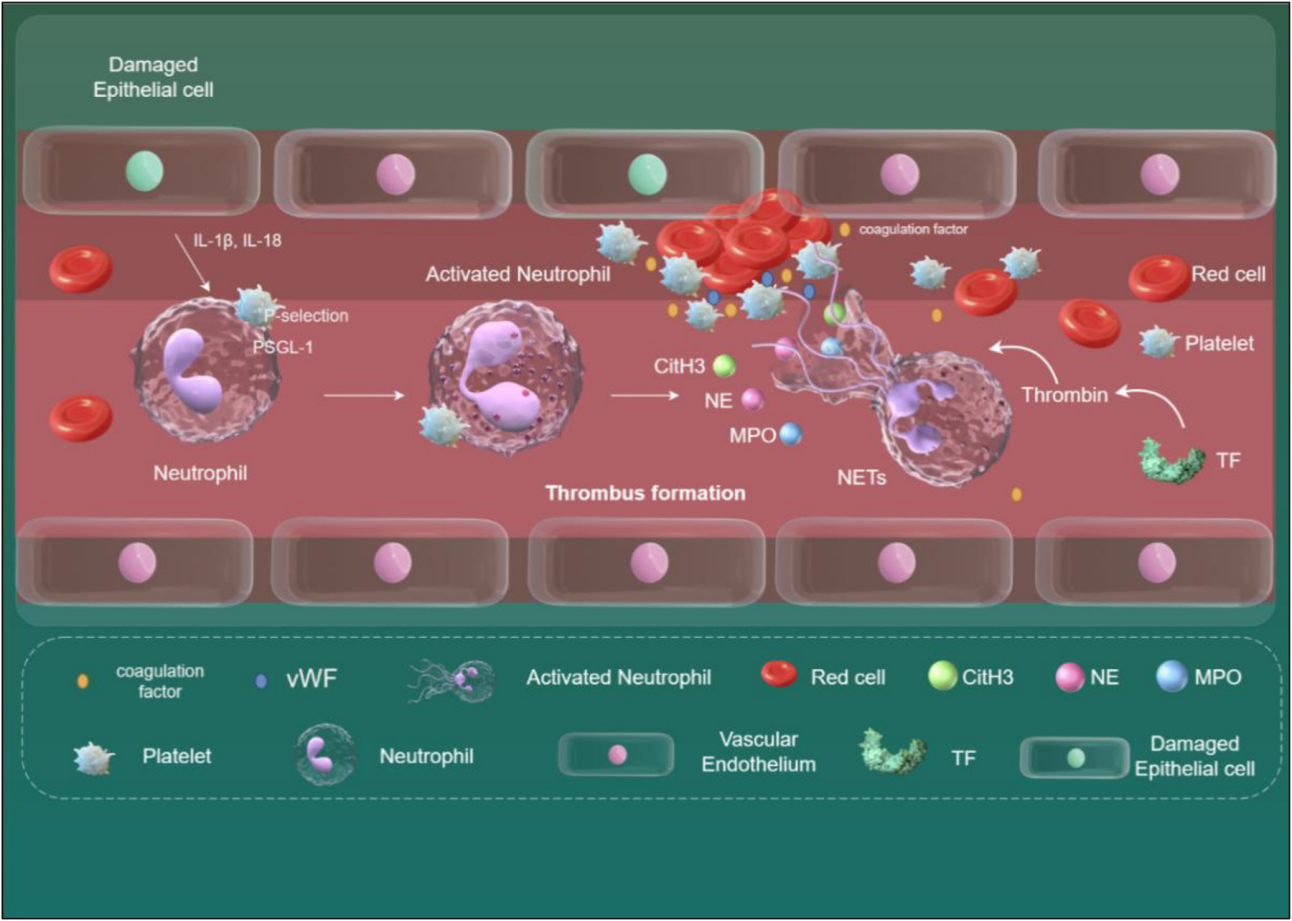
The relationship between NETs and venous thrombosis. When endothelial cells are damaged, they release IL-1β and IL-18, which, in conjunction with other inflammatory factors, activate neutrophils and induce their transformation into NETs. Key mediators triggering NET formation include the binding of HMGB1 on platelets to RAGE on neutrophils, and the binding of P-selectin on platelets to PSGL-1 on neutrophils. NETs can bind to vWF and fibrinogen, providing a scaffold for thrombosis. NETs release various active proteins, including MPO, NE, and CitH3. These proteins disrupt cell adhesion molecules, increase vascular permeability, and lead to endothelial dysfunction. Additionally, the release of TF mediates thrombin formation, further exacerbating thrombosis development. NETs, neutrophil extracellular traps; HMGB1, high-mobility group box protein1; RAGE, receptor for advanced glycation end-products; PSGL-1, P-selectin glycoprotein ligand-1; vWF, von Willebrand factor; MPO, myeloperoxidase; NE, neutrophil elastase; CitH3, citrullinated histone H3; TF, tissue factor. Created using Figdraw.

Platelets and neutrophils form a prothrombotic feedback loop: Platelet-derived P-selectin and chemokines (e.g., CCL5, CXCL4) recruit neutrophils, while P-selectin/PSGL-1 binding triggers NETosis (Figure 3) (111–114). NET components, such as histones, activate platelet NLRP3 inflammasomes, upregulate P-selectin and phosphatidylserine, and enhance thrombin generation, further promoting coagulation (106, 115–117). However, platelet-driven NLRP3 activation in immune cells appears independent of platelet-derived IL-1, suggesting alternative mechanisms like contact-dependent signaling or soluble mediators (118).

While NLRP3's role in platelet-driven arterial thrombosis is well-established, its contribution to VTE remains less defined. Unlike arterial thrombosis, VTE relies more heavily on fibrin-driven mechanisms and leukocyte-mediated coagulation, with platelets playing a secondary role. However, emerging evidence implicates platelet procoagulant activity (PA) in venous thrombosis. Studies show that inhibiting platelet PA reduces venous thrombus formation without compromising trauma-associated hemostasis (64). Platelets promote NET release via GPIbα-neutrophil interactions and support monocyte recruitment. Platelet depletion or GPIbα deficiency (IL4-R/Iba) significantly reduces leukocyte adhesion and venous thrombus size (81). In COVID-19 patients, plasma histones trigger platelet activation, driving platelet-dependent thrombin generation, thrombus growth under venous shear stress, and release of platelet-derived EVs. These EVs enhance thrombin generation *in vitro* and exacerbate venous thrombosis in mice (119). Additionally, neutrophil-endothelial interactions promote platelet aggregation and coagulation factor activation through direct interactions and NET-mediated amplification of clotting responses (120, 121).

In summary, current evidence suggests that compared to the central role of platelets in arterial thrombosis, the functional mechanisms of platelets in VTE remain incompletely elucidated. Although inhibiting platelet activation demonstrates potential clinical value in VTE prevention and treatment, its efficacy is currently limited to adjunctive therapeutic strategies, with further clarification required for its precise molecular mechanisms and therapeutic targets. Additionally, investigations into the functional heterogeneity of platelets in secondary VTE contexts—such as infection, sterile inflammation, and cancer—may provide a theoretical foundation for developing precision antithrombotic therapies.

## 7 Endothelial cells and venous thrombosis

The vascular endothelium indeed plays a crucial role in maintaining homeostasis within the body. Under normal circumstances, it functions to uphold vasodilation and local fibrinolysis, restrain inflammation, and inhibit leukocyte and platelet activation and aggregation, while also facilitating anticoagulant mechanisms (122, 123). However, under stress conditions such as hypoxia or increased oxidative stress, the endothelium can undergo activation and alter its function. This activation can prompt a shift from inhibiting thrombosis to promoting thrombosis and inhibiting fibrinolysis, subsequently triggering an inflammatory response and thrombosis (123, 124). Proinflammatory factors like IL-1β and IL-18 released by endothelial cells following NLRP3 inflammasome activation play a pivotal role in promoting inflammation. This stimulation activates NF-κB signaling, leading to increased expression of chemotactic molecules and adhesion molecules,such as P-selectin and intercellular adhesion molecule-1 (ICAM-1), thereby increased leukocyte adhesion and cell permeability, ultimately resulting in the progression of endothelial dysfunction (125, 126). Furthermore, activated leukocytes secrete inflammatory cytokines such as tumor necrosis factor (TNF) or IL-1, further exacerbating endothelial dysfunction progression (127). Additionally, IL-1β can enhance endothelial expression of procoagulant factors vWF and TF while reducing the expression of endovascular anticoagulant factors (128, 129). The IL-1β-induced endothelial cell surface TF-rich phenotype significantly increases endothelial permeability, promoting thromboinflammation in a positive feedback loop. The local thrombin generated by TF-triggered extrinsic coagulation cascade may contribute to increased endothelial gapping (130). Nonetheless, further *in vivo* experiments are requisite to confirm the specific effects of TF. ROS released during NETs formation can target tight junctions, adherens junctions, and actin filaments in vascular endothelial cells.This oxidative stress damages certain cell signaling proteins, modulates intracellular free calcium concentration,and activates related enzymes, promoting cytoskeleton reorganization and ultimately leading to endothelial barrier disruption and dysfunction (131, 132). NETs also induce TF production on endothelial cell surfaces through mechanisms involving cathepsin and IL-1α, thus eliciting a thromboinflammatory response (133, 134). Moreover, histones can facilitate pro-inflammatory protein scaffolds by interacting with endothelial cells, thereby contributing to inflammatory processes (88). In addition, IL-1β and NETs play crucial roles in promoting the exocytosis of endothelial Weibel-Palade bodies (WPBs). IL-1β, as a key pro-inflammatory cytokine, can influence WPB exocytosis through multiple mechanisms. Studies have shown that IL-1β can enhance WPB exocytosis by activating the NF-κB signaling pathway, thereby promoting inflammatory responses in endothelial cells (74, 135). Furthermore, IL-1β can directly stimulate WPB exocytosis through binding to its receptor, leading to the release of ultra-large von Willebrand factor (UL-VWF), ICAM-1, and P-selectin (136). NETs can indirectly augment WPB exocytosis by promoting inflammatory responses in vascular endothelial cells (137). Additionally, histones within NETs can directly induce WPB exocytosis, thereby facilitating the release of UL-VWF and other pro-inflammatory factors (137). Under pathological conditions such as myocardial infarction, the activation of the NLRP3 inflammasome and the formation of NETs can significantly exacerbate myocardial injury, a process closely associated with IL-1β secretion and WPB exocytosis (138). Therefore, targeting IL-1β and NETs may serve as a critical therapeutic strategy to mitigate inflammatory responses in cardiovascular diseases (136, 138). Under low-shear venous conditions, UL-VWF multimers form platelet-decorated strings (139). Under physiological conditions, ADAMTS13 cleaves UL-VWF to prevent excessive thrombus formation (140). Oxidants such as hypochlorous acid (HOCl) released by neutrophils can induce the loss of ADAMTS13 activity by oxidizing critical methionine residues within the enzyme, thereby leading to abnormal accumulation of VWF multimers (141).

Alternatively, activated endothelial cells may release extracellular vesicles (EVs) containing cellular contents that play roles in regulating inflammation, coagulation, and leukocyte recruitment. These EVs, particularly those carrying coagulation factors in the bloodstream, can serve as biomarkers for thrombotic risk in certain clinical contexts (142, 143). In a study, it was found that the release of monocyte-derived microparticles (a subset of EVs) can activate endothelial cells via the NLRP3 inflammasome, leading to endothelial dysfunction (74). Additionally, activation of the NLRP3 inflammasome triggers the release of HMGB1, intensifying the contractile activity and permeability of endothelial cells (144). Concurrently, studies have found that nicotine can impair endothelial intercellular junctions and induce endothelial barrier dysfunction by activating the cathepsin B-dependent NLRP3 inflammasome to release HMGB1 (145). In a murine model of acute lung injury, deficiency of NLRP3 has been found to reduce vascular permeability (146). These studies suggest a correlation between the activation of the NLRP3 inflammasome and the induction of endothelial dysfunction at certain levels.

These studies suggest a correlation between the activation of NLRP3 inflammasome and the induction of endothelial dysfunction, yet further exploration is warranted to delineate the specific mechanisms and clinical implications.

## 8 NLRP3 inflammasome: thrombosis timeline

In the inflammatory process, the activation of the NLRP3 inflammasome and venous thrombosis involve dynamic interactions among macrophages, neutrophils, platelets, and endothelial cells. In the initial phase, macrophages recognize PAMPs or DAMPs through TLRs, activating the NF-κB pathway and initiating the transcription of NLRP3 and pro-IL-1β (147). Endothelial cells, under the influence of inflammatory factors such as TNF-α, express adhesion molecules (ICAM-1, VCAM-1) to promote leukocyte recruitment (148), while platelets are initially activated upon contact with damaged endothelium or collagen, releasing ADP and TXA2 (149). In the early phase, macrophages, triggered by secondary signals such as ATP, ROS, and K^+^ efflux, assemble the NLRP3 inflammasome, activate caspase-1, cleave pro-IL-1β/IL-18, induce pyroptosis, and release DAMPs such as HMGB1 (10). Endothelial cells, under the influence of IL-1β, upregulate TF and vWF, promoting platelet adhesion and thrombin generation. Neutrophils are recruited to the inflammatory site by IL-8, initiating phagocytosis and ROS release (150). During the peak phase, neutrophils release NETs, which activate FXII and platelets through histones and DNA, directly promoting thrombosis (151). Platelets aggregate with activated endothelium and NETs, releasing pro-inflammatory factors such as PF4 and CD40l to feedback-activate macrophages (152). Endothelial cells continuously express TF and vWF, inhibit anticoagulant mechanisms such as thrombomodulin, and promote fibrin deposition (153). NLRP3, through IL-1β, induces TF expression and inhibits fibrinolysis (upregulating PAI-1), forming “immunothrombosis” (154). During the late-phase resolution of venous thromboinflammation, controlled inflammation leads to IL-10-mediated NLRP3 inhibition, M2 macrophage polarization, endothelial anticoagulant function restoration, and tPA-dependent fibrin clearance (155). TGF-β cooperates with IL-10 to suppress NLRP3 activation, demonstrating context-dependent functions, facilitating transient extracellular matrix repair in acute DVT resolution (156, 157), yet promoting SMAD3-dependent fibrosis through α-SMA + myofibroblast activation in chronic post-thrombotic syndrome, ultimately resulting in venous wall stiffening and valvular dysfunction (158, 159). If inflammation persists, sustained NLRP3 activation leads to endothelial injury and excessive platelet activation, promoting DVT (160).

## 9 Analysis of NLRP3 inflammasome and thrombosis mechanisms

A growing body of evidence highlights the pivotal regulatory role of the NLRP3 inflammasome in thrombosis. This section systematically reviews studies elucidating the mechanisms by which inflammasome activation mediates venous thrombosis, including its interplay with coagulation cascades and inflammatory signaling, and evaluates emerging therapeutic strategies targeting inflammasome pathway components (Table 1).

**Table 1:** Investigating the impact of targeted NLRP3 inflammasome pathway inhibition on venous thrombosis development.

Model	Target inhibition	Research findings/conclusions	References
Rat IVC stenosis	NLRP3 siRNA Caspase-1 inhibitor IL-1β antibody	1. The use of NLRP3 siRNA, caspase-1 inhibitors, and IL-1β-specific antibodies significantly reduced thrombus formation, decreased platelet aggregation, and lowered coagulation parameters in rats.	(161)
Mice IVC stenosis	Entpd1^−^/^−^ IL-1 receptor antagonist (Anakinra) IL-1β neutralizing antibody	1. Entpd1^−^/^−^ mice exhibited a significant increase in thrombus weight after IVC ligation. 2. Administration of an IL-1 receptor antagonist (Anakinra) or IL-1β neutralizing antibody significantly reduced thrombus formation in Entpd1^−^/^−^ mice.	(162)
Mice IVC stenosis	NLRP3^−^/^−^ Caspase-1/11^−^/^−^ GSDMD^−^/^−^	1. NLRP3^−^/^−^, Caspase-1/11^−^/^−^, and GSDMD^−^/^−^ mice showed a significant reduction in thrombus weight or volume compared to wild-type mice.	(79)
Mice IVC stenosis	NLRP3^−^/^−^ PAD4^−^/^−^	1. PAD4 serves as a critical auxiliary factor for NLRP3 inflammasome assembly and drives NETosis. 2. NETosis exacerbates sterile inflammation and promotes thrombus formation.	(97)
Mice IVC stenosis	caspase-1 inhibitor	1. NETs activate caspase-1 in platelets, promoting the formation of venous thrombosis in mice. 2. Administration of a selective caspase-1 inhibitor significantly reduces thrombosis formation in mice.	(116)
Rat IVC stenosis	HIF-1α inhibitor Res HIF-1α inhibitor+Res	1. Resveratrol effectively inhibits thrombus formation, reduces coagulation parameters, and downregulates the expression levels of IL-1β, caspase-1, HIF-1α, and NLRP3.	(163)
Rat IVC stenosis	si-TXNIP NLRP3 inhibitor (MCC950)	1. Inhibition of TXNIP or administration of the NLRP3 inhibitor MCC950 significantly reduced thrombus weight, coagulation parameters, and levels of inflammatory cytokines (IL-1β, IL-18).	(164)

Gupta N et al. demonstrated that hypoxic conditions in rats induced significant venous thrombosis alongside enhanced NLRP3 inflammasome activity. Administration of HIF inhibitors or HIF-1α siRNA reduced NLRP3/IL-1β expression and attenuated thrombus formation (161). NLRP3 siRNA, caspase-1 inhibitors, or IL-1β-neutralizing antibodies significantly decreased thrombus weight, coagulation parameters, and platelet aggregation (161). In high-altitude thrombosis patients (without traditional thrombotic risk factors), peripheral blood monocytes exhibited elevated NLRP3, caspase-1, IL-1β, and IL-18 expression, with increased plasma NLRP3 protein and caspase-1 activity, consistent with animal models (161). Targeting HIF-1α or NLRP3 inflammasome components (e.g., inhibitors or gene silencing) may represent novel strategies for preventing or treating hypoxia-associated venous thrombosis, particularly in high-altitude populations or patients with chronic hypoxia-related diseases (e.g., COPD, sleep apnea).

Yadav V et al. investigated the role of ectonucleoside triphosphate diphosphohydrolase 1 (ENTPD-1) in suppressing venous thrombosis via NLRP3 inflammasome and IL-1β inhibition (162). Results showed that ENTPD-1 deficiency promoted NET formation, exacerbated NLRP3 inflammasome activation, and increased IL-1β release, thereby accelerating thrombosis (162). IL-1β-neutralizing antibodies or IL-1 receptor antagonists markedly reduced thrombus formation (162). Targeting CD39 (ENTPD-1) or its downstream effectors (e.g., IL-1β) may offer novel therapeutic approaches for venous thrombosis, particularly in refractory deep vein thrombosis (DVT) or individuals with chronic inflammation or inherited CD39 deficiency.

Zhang et al. explored pyroptosis-driven venous thrombosis via inflammasome activation (79). Caspase-1^−^/^−^ and GSDMD^−^/^−^ mice exhibited significantly reduced thrombus formation and fibrin deposition, whereas Caspase-11^−^/^−^ mice showed no notable differences in thrombosis parameters, indicating the predominance of the canonical inflammasome pathway in venous thrombosis (79). Monocyte/macrophage depletion via gadolinium chloride or tissue factor (TF) deficiency also prevented thrombosis. Targeting Caspase-1 or GSDMD (e.g., inhibitors) may provide novel interventions for venous thrombosis, potentially reducing bleeding risks associated with conventional anticoagulants (79). This study highlights the Caspase-1/GSDMD axis in monocyte/macrophage pyroptosis-mediated TF release, contrasting with prior findings implicating NLRP3-driven IL-1β in thrombosis, suggesting context-dependent roles of inflammasomes in distinct pathological settings.

Münzer P et al. focused on NLRP3 inflammasome assembly in neutrophils and the auxiliary role of PAD4 (97). Their study revealed PAD4 deficiency impaired NLRP3 inflammasome assembly and reduced NET release, with diminished NLRP3 and ASC activation (97). Importantly, NLRP3−/− mice or MCC950 (NLRP3 inhibitor)-treated mice showed reduced NETosis and venous thrombosis (97). These findings identify PAD4 as a critical regulator of NLRP3 inflammasome activation and NET-dependent thrombosis. Future studies should explore whether PAD4 functions synergistically with cytokines, enzymes, or metabolites to modulate inflammasome activity. Emerging evidence underscores the NLRP3–NET axis as a conserved mechanism across sterile inflammatory diseases. A recent study utilizing a permanent left anterior descending (LAD) artery ligation model of acute myocardial infarction (AMI) demonstrated that NLRP3 activation exacerbates disease progression by amplifying NET-driven microvascular occlusion and tissue damage, which aligns with evidence of NLRP3-dependent NETosis in post-infarction neutrophils (138). These NETs not only directly obstruct capillaries but also expose proteases and proinflammatory molecules that amplify local inflammation and recruit additional immune cells (138). The resulting positive feedback loop between NLRP3 activation and NETosis perpetuates ischemic injury, a pathological paradigm that can be therapeutically targeted via NLRP3 inhibition or NET disruption strategies (138). Mechanistically, NLRP3 inflammasome assembly in neutrophils triggers histone citrullination and chromatin decondensation to drive NET release. While this process is regulated by peptidylarginine deiminase 4 (PAD4) activity, the precise regulatory interplay between them remains incompletely characterized.

Campos et al. investigated the synergistic role of NETs and inflammasomes in murine DVT (116). Activated caspase-1 in thrombus-associated platelets and leukocytes colocalized with NETs (116). Histones (particularly H3/H4) activated caspase-1 in human platelets, and coculture with NETotic neutrophils amplified caspase-1 activity, suggesting NET-derived histones drive platelet inflammasome activation (116). Caspase-1 inhibitors reduced thrombus formation and citrullinated histone H3 (Cit-H3, a NET marker) levels, implying caspase-1 inhibition may exert antithrombotic effects by suppressing NETosis (116). Targeting caspase-1 or NET components (e.g., histone-neutralizing antibodies) may offer safer alternatives to IL-1β inhibitors (e.g., canakinumab) by minimizing infection risks.

Fei et al. demonstrated that resveratrol (Res) inhibits venous thrombosis via the HIF-1α/NLRP3 axis. Res reduced thrombus formation and suppressed HIF-1α, NLRP3, IL-1β, and caspase-1 expression (163). HIF-1α inhibitors or Res alone attenuated thrombosis, while combination therapy further enhanced antithrombotic efficacy, confirming HIF-1α as Res's primary target (163). As a natural polyphenol with high safety, Res holds promise for DVT prevention or adjunct therapy (163). In a separate study, Fei et al. identified thioredoxin-interacting protein (TXNIP) as a key regulator of DVT via NLRP3 activation and oxidative stress (164). TXNIP and NLRP3 expression correlated with thrombus weight, while thioredoxin (TRx) declined (164). TXNIP inhibition or MCC950 reduced thrombosis, inflammatory cytokines (IL-1β, IL-18), and oxidative stress markers (e.g., elevated MDA, reduced SOD/GSH-Px) (164). These findings reveal TXNIP's dual role in NLRP3 activation and oxidative damage, suggesting therapeutic potential in targeting this pathway. However, TXNIP may also engage NLRP3-independent mechanisms in DVT, warranting further investigation.

## 10 NLRP3 inhibitors: therapeutic potential of pharmacological NLRP3 inflammasome inhibition in thrombotic disorders

Thrombotic diseases, such as deep vein thrombosis and atherosclerosis-related thrombosis, are closely associated with chronic inflammation in their pathological progression. The NLRP3 inflammasome, a central regulatory hub of the innate immune system, has emerged as a critical therapeutic target in pro-thrombotic inflammatory cascades by driving IL-1β/IL-18 release, NET formation, and endothelial cell pyroptosis. This section will introduce several potential therapeutic agents for venous thromboembolic diseases.

### 10.1 MCC950

MCC950, a molecular inhibitor, selectively blocks NLRP3 ATP hydrolysis activity to inhibit the assembly and activation of the NLRP3 inflammasome, thereby effectively suppressing the maturation and release of IL-1β and IL-18. Experimental studies demonstrate that this compound significantly alleviates pathological damage in both *in vitro* models and diverse murine inflammatory disease models (including autoinflammatory disorders and multiple sclerosis), without interfering with other inflammatory pathways. Consequently, MCC950 has garnered widespread consideration for therapeutic applications in NLRP3-associated inflammatory diseases (165). In studies conducted by Fei et al., MCC950 administration markedly inhibited venous thrombus formation and reduced pro-inflammatory cytokine production (e.g., IL-1β), highlighting its dual anti-inflammatory and antithrombotic efficacy (164).

### 10.2 CY-09

CY-09, a novel NLRP3 inhibitor, selectively blocks NLRP3 inflammasome assembly and downstream IL-1β/IL-18 release by directly binding to the NACHT domain of NLRP3 and inhibiting its ATPase activity. Experimental validation demonstrates that CY-09 effectively alleviates pathological damage in both *in vitro* macrophage models and murine inflammatory models, including gouty arthritis and sepsis, without interfering with other inflammasome pathways such as AIM2 or NLRC4 (166). Furthermore, this study reveals that NLRP3 enhances platelet spreading, fibrinogen binding, and thrombus stability by regulating integrin αIIbβ3-mediated outside-in signaling. By inhibiting NLRP3, CY-09 selectively suppresses pathological arterial thrombosis while preserving physiological hemostasis. This targeted inhibition suggests that the NLRP3-αIIbβ3 axis may offer a novel therapeutic strategy for antithrombotic treatment, effectively reducing thrombosis without increasing bleeding risk (104).

### 10.3 Colchicine

Colchicine indirectly suppresses NLRP3 inflammasome activation by binding to β-tubulin, thereby inhibiting microtubule polymerization and promoting their depolymerization (167). This microtubule disruption impairs the microtubule-organizing center (MTOC)-dependent assembly of NLRP3 inflammasome components, such as ASC speck formation (167). Importantly, colchicine's mechanism of action is not specific to the NLRP3 inflammasome. As a broad microtubule-disrupting agent, it interferes with diverse microtubule-dependent cellular processes, including neutrophil migration, vesicular trafficking, and cytokine secretion. This non-specificity explains both its pleiotropic anti-inflammatory effects and dose-limiting toxicities—such as gastrointestinal disturbances (e.g., diarrhea) and hematological complications (e.g., myelosuppression)—which are closely linked to systemic microtubule dysfunction (167). Despite its lack of target specificity, colchicine has demonstrated significant clinical benefits in cardiovascular diseases. In the LoDoCo2 trial, low-dose colchicine (0.5 mg/day) reduced major adverse cardiovascular events by 31% in chronic coronary artery disease patients, with a safety profile supporting long-term use (168). Notably, its therapeutic efficacy in complex diseases like atherosclerosis likely stems from simultaneous modulation of multiple microtubule-dependent pathways (e.g., leukocyte recruitment, inflammasome activation, and endothelial dysfunction) (169, 170). Experimental studies further suggest its potential in α-Def-1-mediated thrombosis models (171). Although the molecular mechanisms of colchicine in venous thrombosis remain uncharacterized, its efficacy in arterial and α-Def-1-mediated thrombosis models suggests potential therapeutic utility for venous thrombotic disorders.

### 10.4 OLT1177

OLT1177 (Dapansutrile), a safe and effective NLRP3 inflammasome inhibitor, significantly suppresses IL-1β/IL-18 release by blocking ASC oligomerization and caspase-1 activation. In murine inflammatory models, such as monosodium urate-induced acute gout, OLT1177 reverses inflammation-associated energy metabolism imbalances. Phase I clinical trials have demonstrated its excellent tolerability in healthy volunteers, along with its ability to reduce systemic inflammatory markers (e.g., CRP) and improve mitochondrial respiratory function. These findings suggest that OLT1177 reduces the “inflammatory metabolic cost” (i.e., excessive energy expenditure caused by chronic inflammation) through NLRP3 pathway inhibition, offering a novel therapeutic strategy for NLRP3-driven metabolic-inflammatory comorbidities, such as gout and type 2 diabetes (172). Furthermore, OLT1177 has exhibited potent anti-inflammatory effects in gout, intracerebral hemorrhage-induced brain injury, and spinal cord injury, effectively controlling disease progression (173, 174). Although research on its role in thrombosis remains limited, OLT1177 represents a promising therapeutic candidate, warranting further investigation into its potential efficacy in thrombotic disorders.

### 10.5 Tranilast

Tranilast, an anti-allergic drug, exerts its effects by directly binding to the NACHT domain of the NLRP3 inflammasome, thereby inhibiting its ATPase activity and oligomerization, which blocks the release of IL-1β and IL-18. Its specific anti-inflammatory action has been validated in murine models of gouty arthritis and peritonitis, demonstrating selective targeting of NLRP3 without affecting other inflammasomes (166). Studies reveal that Tranilast promotes ubiquitination of the NLRP3 protein, accelerating its proteasomal degradation, which subsequently suppresses the expression of vascular inflammation markers such as VCAM-1 and ICAM-1. In an atherosclerosis model (ApoE^−^/^−^ mice), Tranilast significantly reduces plaque area. Through its dual mechanisms of “direct NLRP3 inhibition” and “regulation of protein stability,” Tranilast provides multidimensional evidence supporting the repurposing of this drug for treating NLRP3-driven cardiovascular and immune disorders (175). Although research on its inhibitory effects in venous thrombosis remains limited, the drug's mechanistic profile and preliminary findings suggest potential therapeutic utility in venous thrombosis, highlighting its broader applicability in thromboinflammatory conditions.

### 10.6 Anakinra

Anakinra (IL-1 receptor antagonist) demonstrates dual therapeutic value in inflammatory diseases:For colchicine-resistant and corticosteroid-dependent recurrent pericarditis (AIRTRIP trial), anakinra treatment induced acute-phase remission in 90% of patients, with significantly lower recurrence rates in the maintenance group compared to placebo (18.2% vs. 90%, *P* < 0.001) and a median relapse-free survival exceeding 14 months. The main adverse effects were reversible skin reactions (95.2%) and mild liver enzyme elevation (14.3%) (176). In severe COVID-19 patients, high-dose intravenous anakinra significantly reduced thrombotic events compared to standard of care (5% vs. 12.3%, OR = 4.3), particularly pulmonary embolism (2.9% vs. 9.6%) and acute coronary syndrome risk, with thrombosis strongly linked to hyperinflammation (177). These studies collectively suggest that targeting the IL-1 pathway not only controls organ-specific inflammation but also improves outcomes by modulating systemic thromboinflammatory responses. However, opportunistic infection risks must be monitored, and biomarker-based patient stratification is needed to optimize benefits. Larger-scale studies are still required to confirm long-term efficacy.

### 10.7 Canakinumab

The CANTOS trial provided the first clinical evidence that anti-inflammatory therapy can reduce cardiovascular risk: In 10,061 post-myocardial infarction patients with elevated high-sensitivity C-reactive protein, the IL-1β monoclonal antibody canakinumab significantly reduced inflammatory markers (26%–41% greater hs-CRP reduction vs. placebo) without affecting lipid levels. After a median follow-up of 3.7 years, only the 150 mg dose group met prespecified statistical significance thresholds, demonstrating a 15% reduction in the primary endpoint (nonfatal myocardial infarction/stroke/cardiovascular death) and a 17% reduction in the composite endpoint including unstable angina requiring revascularization compared to placebo. However, canakinumab was associated with increased fatal infections (higher incidence than placebo) while showing no significant difference in all-cause mortality (178). This study proved that IL-1β pathway inhibition can achieve cardiovascular secondary prevention independent of lipid-lowering, but requires careful risk-benefit assessment regarding infection risks, highlighting the need for optimized dosing and patient stratification in targeted anti-inflammatory therapy.

### 10.8 Entrectinib

Entrectinib (ENB) is an orally bioavailable small-molecule tyrosine kinase inhibitor initially developed for treating NTRK fusion-positive solid tumors and ROS1-positive non-small cell lung cancer (NSCLC). By targeting specific tyrosine kinase receptors, ENB demonstrates significant antitumor activity and favorable tolerability (179, 180). Recent studies have revealed that Entrectinib serves as a specific inhibitor of the NEK7-NLRP3 interaction, effectively blocking the NLRP3 inflammasome pathway. Mechanistically, ENB directly binds to arginine 121 (R121) of NEK7, disrupting the NEK7-NLRP3 interaction and thereby suppressing inflammasome assembly and activation. Notably, ENB selectively targets NLRP3 without affecting other inflammasomes (e.g., AIM2, NLRC4).In multiple disease models—including LPS-induced systemic inflammation, MSU-induced peritonitis, and high-fat diet (HFD)-induced type 2 diabetes (T2D)—ENB exhibits potent anti-inflammatory and therapeutic effects (181). These findings suggest that ENB, as the first NEK7-targeting NLRP3 inhibitor, holds promise for drug repurposing in inflammatory diseases.

### 10.9 Comparative perspectives and challenges

Current NLRP3 inhibitors exhibit diverse mechanisms and clinical applicability (Table 2). MCC950 and CY-09 represent the most specific NLRP3-targeting agents, directly blocking ATPase activity with minimal off-target effects, but their clinical translation is limited by safety concerns (e.g., MCC950 hepatotoxicity) or insufficient thrombosis-specific data. Colchicine, despite its non-specificity, remains a practical option due to its proven efficacy in cardiovascular trials and low cost, albeit requiring careful monitoring of side effects. Biologics (Anakinra, Canakinumab) provide potent IL-1 pathway suppression but carry infection risks and high costs, restricting their use to severe inflammatory conditions. Entrectinib uniquely targets the NEK7-NLRP3 interaction, combining antitumor and anti-inflammatory effects, yet its role in thrombosis warrants further exploration. Future efforts should prioritize developing NLRP3 inhibitors with enhanced specificity, oral bioavailability, and validated safety profiles for thromboinflammatory diseases.

**Table 2:** Comparative analysis of NLRP3 inhibitors in thrombotic and inflammatory diseases.

Inhibitor	Mechanism of action	Specificity for NLRP3	Key advantages	Limitations/side effects	Clinical stage
MCC950	Directly inhibits NLRP3 ATPase activity	High	Highly selective; no interference with AIM2/NLRC4 Dual anti-inflammatory and antithrombotic effects	Clinical trials paused due to hepatotoxicity	Preclinical/Phase I (discontinued)
CY-09	Binds NLRP3 NACHT domain, blocks ATPase	High	Preserves physiological hemostasis No bleeding risk Oral bioavailability	Limited long-term safety data	Preclinical
Colchicine	Indirectly inhibits NLRP3 via microtubule depolymerization	Low	Clinically validated in CVD Low-cost Oral administration	Non-specific (affects all microtubule pathways) GI toxicity, myelosuppression	Approved (off-label use)
OLT1177	Blocks ASC oligomerization	Moderate	Improves mitochondrial function Favorable safety profile	Limited thrombosis-specific data	Phase II (gout, COVID-19)
Tranilast	Promotes NLRP3 ubiquitination and degradation	Moderate	Dual mechanism (inhibition + degradation) Reduces plaque area in atherosclerosis	Limited clinical data in thrombosis	Preclinical/Repurposing
Anakinra	IL-1 receptor antagonist	Low	Rapid anti-inflammatory effects Proven efficacy in recurrent pericarditis	Immunosuppression (infection risk) Short half-life	Approved (autoimmune diseases)
Canakinumab	Monoclonal antibody against IL-1β	Low	Long-acting (q3-month dosing) CANTOS trial validation	High cost Fatal infection risk	Approved (CAPS, off-label CVD)
Entrectinib	Disrupts NEK7-NLRP3 interaction	High	Oral bioavailability Dual antitumor/anti-inflammatory effects	Tyrosine kinase inhibition (off-target) Limited thrombosis data	Approved (cancer), Preclinical repurposing

## 11 Conclusion and prospective outlook

In recent years, the NLRP3 inflammasome has been widely studied as an important immune sensor, playing a crucial role in various diseases. In thrombosis and related cardiovascular diseases, activation of the NLRP3 inflammasome is considered a key mechanism in regulating thrombosis. A deeper understanding of the relationship between the NLRP3 inflammasome and thrombosis is important for elucidating the pathogenesis of thrombotic diseases and developing new therapeutic strategies. However, current research still faces some limitations. Most existing studies rely on animal models or cell-based experiments, which provide preliminary biological insights but lack large-scale human clinical data. These models may not fully reflect human physiology, and interspecies differences may limit the generalizability of the results. Particularly in the complex physiological process of thrombosis, the interaction of multiple factors makes it difficult for studies focusing on a single mechanism to fully reveal its nature. Additionally, NLRP3 inflammasome inhibitors such as MCC950 have shown promising anti-thrombotic effects, but their long-term safety has not been fully evaluated. Since NLRP3 plays a crucial role in immune defense, its inhibition may lead to immunosuppression, increasing the risk of infections and other complications. This is especially concerning for the elderly, immunocompromised individuals, and patients with multiple comorbidities, as excessive inhibition of NLRP3 may have adverse consequences. Future research should focus on how the NLRP3 inflammasome promotes thrombosis through immune-coagulation interactions. Specifically, understanding how NLRP3 regulates endothelial cell function, platelet aggregation, and leukocyte migration will provide new insights into thrombosis prevention and treatment.

At present, research on NLRP3 as a thrombosis-related biomarker is still in its early stages, with insufficient clinical validation. Future studies should conduct large-scale, multi-center clinical trials to verify whether the NLRP3 inflammasome can serve as an early diagnostic biomarker or risk assessment tool for VTE. These studies should comprehensively consider factors such as patients' genetic backgrounds, levels of inflammation, and hemodynamics, to explore personalized treatment strategies. While NLRP3 inflammasome inhibitors have entered preclinical stages, their application in thrombosis treatment still faces significant challenges. Future research should conduct more in-depth pharmacological and clinical studies to assess the immunosuppressive effects, long-term safety, and tolerability of these inhibitors. Furthermore, precision medicine could help identify the most suitable patient populations for targeted therapies, while minimizing unnecessary side effects, which will be a key focus of future studies. The relationship between the NLRP3 inflammasome and thrombosis may vary across individuals, making personalized treatment particularly important. In the future, combining genomics and proteomics may help identify susceptible populations, allowing for the development of tailored treatment plans. For example, differences in gene expression profiles may determine the level of NLRP3 activation in different patients, influencing the risk of thrombosis and response to treatment.
